# Ultrasonographic diagnosis of erythema nodosum

**DOI:** 10.1111/srt.13112

**Published:** 2021-11-23

**Authors:** Gianluca Nazzaro, Carlo Alberto Maronese, Emanuela Passoni

**Affiliations:** ^1^ Dermatology Unit Fondazione IRCCS Ca' Granda Ospedale Maggiore Policlinico Milan Italy; ^2^ Department of Pathophysiology and Transplantation Università degli Studi di Milano Milan Italy

**Keywords:** erythema, nodosum, panniculitis, ultrasound

To the Editor,

Erythema nodosum (EN) is an acute, septal panniculitis, presumably resulting from massive deposition of immune complexes in the venules of subcutaneous fat.^1^ It represents a stereotypical reaction pattern to a wide variety of triggering factors ‐ such as infections, autoimmune diseases, drugs, and classically manifests with painful, rounded to oval, slightly raised, non‐ulcerative erythematous nodules on the extensor aspect of the lower limbs.^1^

Although the execution of a biopsy may be spared in classic cases, due to its particularly painful nature and the intrinsic risk of sequelae, often obtaining histopathological confirmation is unavoidable. Noninvasive assessment of EN lesions represents a partially unmet need in the management of this condition. More specifically, the available evidence regarding EN sonographic features appears to be limited.

Herein, we present a five‐patient case series, illustrating corresponding ultrasonographic and histopathological findings of EN and corroborating the role of US in the approach to EN.

Demographics, clinical and ultrasonographic features are summarized in Table 1.

**TABLE 1:** Patient characteristics

Patient	Sex, age	History	Clinical features	US features	Histopathological features
**Patient 1**	**F, 42**	**HS, IBD**	Pretibial tender nodosity, with accompanying erythema	Thickened, hyperechoic, not easily compressible subcutaneous tissue. Septal inflammation with an increased blood flow on Doppler mode	Septal panniculitis
**Patient 2**	**F, 82**	**NA**	Tender, subcutaneous nodosity on the patient's right abdominal region.	Thickened, hyperechoic subcutaneous tissue, with prominent hypoechoic septa.	Septal panniculitis
**Patient 3**	**M, 36**	**Polymyositis**	Bilateral pretibial erythema, with tender, subcutaneous nodosity on palpation.	Thickened, hyperechoic subcutaneous tissue, with blurry hypoechoic septa. Mixed septal/lobular panniculitis.	Septal panniculitis with foci of lobular involvement.
**Patient 4**	**F, 52**	**NA**	Bilateral pretibial erythema, with tender, subcutaneous nodosity on palpation.	Thickened, hyperechoic, not easily compressible subcutaneous tissue. Septal inflammation with an increased blood flow on Doppler mode	Septal panniculitis
**Patient 5**	**M, 30**	**NA**	Subcutaneous, tender lesions on the right calf. Clinically mistaken as an organized hematoma.	Diffusely hyperechoic subcutaneous tissue, with hypoechoic, focally blurred, septa.	Septal panniculitis

Abbreviation: HS, hidradenitis suppurativa; IBD, inflammatory bowel disease; NA, not available.

Cutaneous ultrasonography (ARIETTA 850, Hitachi Medical Systems, Zug, Switzerland; multifrequency 15.0–18.0 MHz linear array transducer) was performed in order to ascertain the depth and the extent of the ongoing inflammatory process and to better navigate the differential diagnosis.

In all patients, marked hyperechogenicity of subcutaneous fat lobules was appreciated, as well as the presence of non‐compressible, hypoechoic septa, with an increased vascular flow on Doppler evaluation (Figure 1). In one patient, septal structures appeared to be blurrier, with hyperechoic subcutaneous lobules almost blending together, at least focally (Figure 2). Overall, the ultrasonographic picture oriented toward a deep inflammatory process showing predominant involvement of the subcutaneous septa. The absence of vasculitis favored a presumptive diagnosis of EN.

**FIGURE 1:**
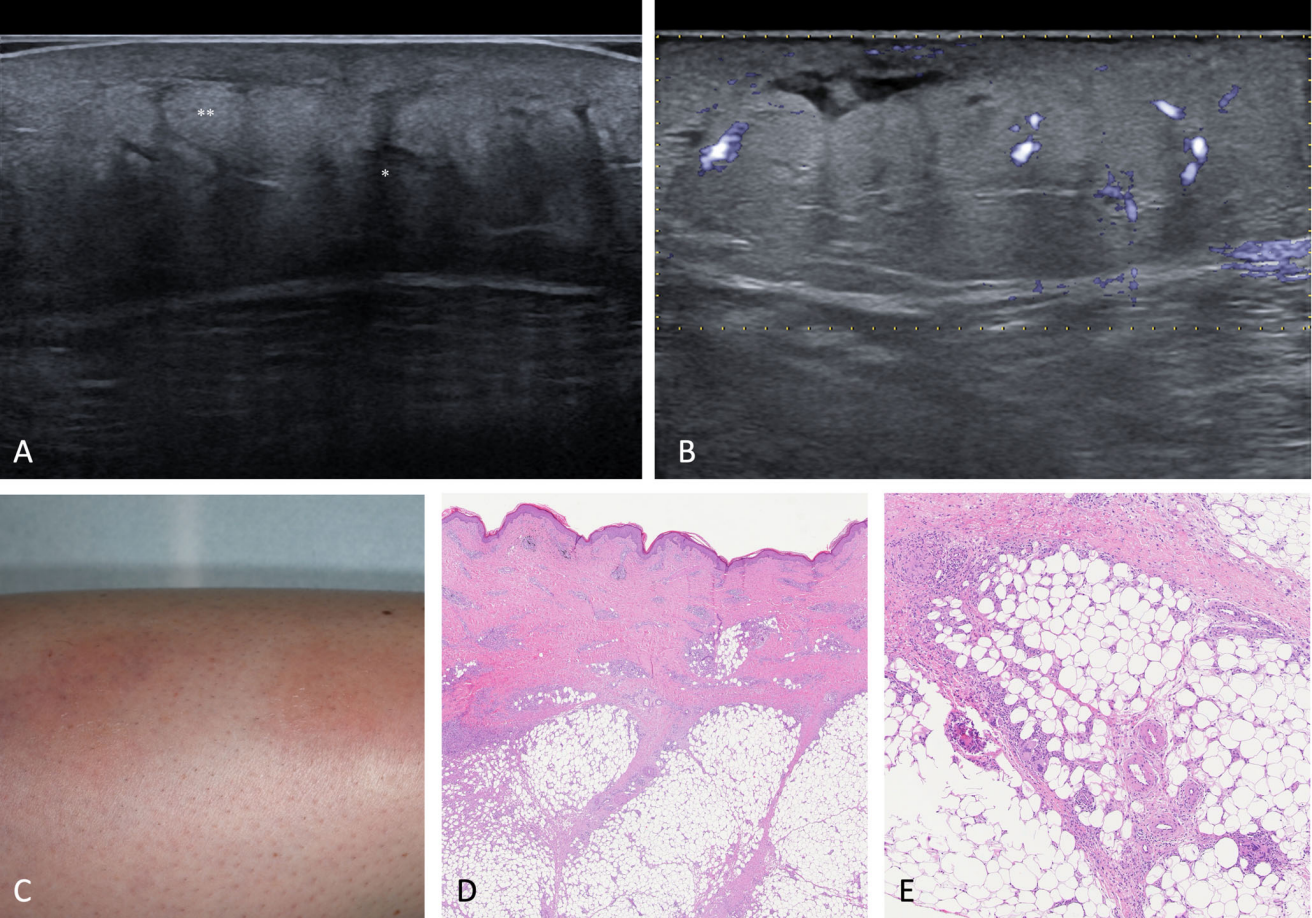
Septal panniculitis. (A) Ultrasonographic features of septal panniculitis with a typical “jigsaw” pattern. Notice the hypoechoic, thickened septa (*) and hyperechoic lobules (**). (B) Increased vascular flow on Doppler evaluation. (C) Clinical presentation of erythema nodosum (EN), with slightly raised, non‐ulcerative erythematous nodules on the anterior surface of the lower limbs. (D) Histopathological features of EN (H&E, 20x). (E) Higher magnification showing (H&E, 200x)

**FIGURE 2:**
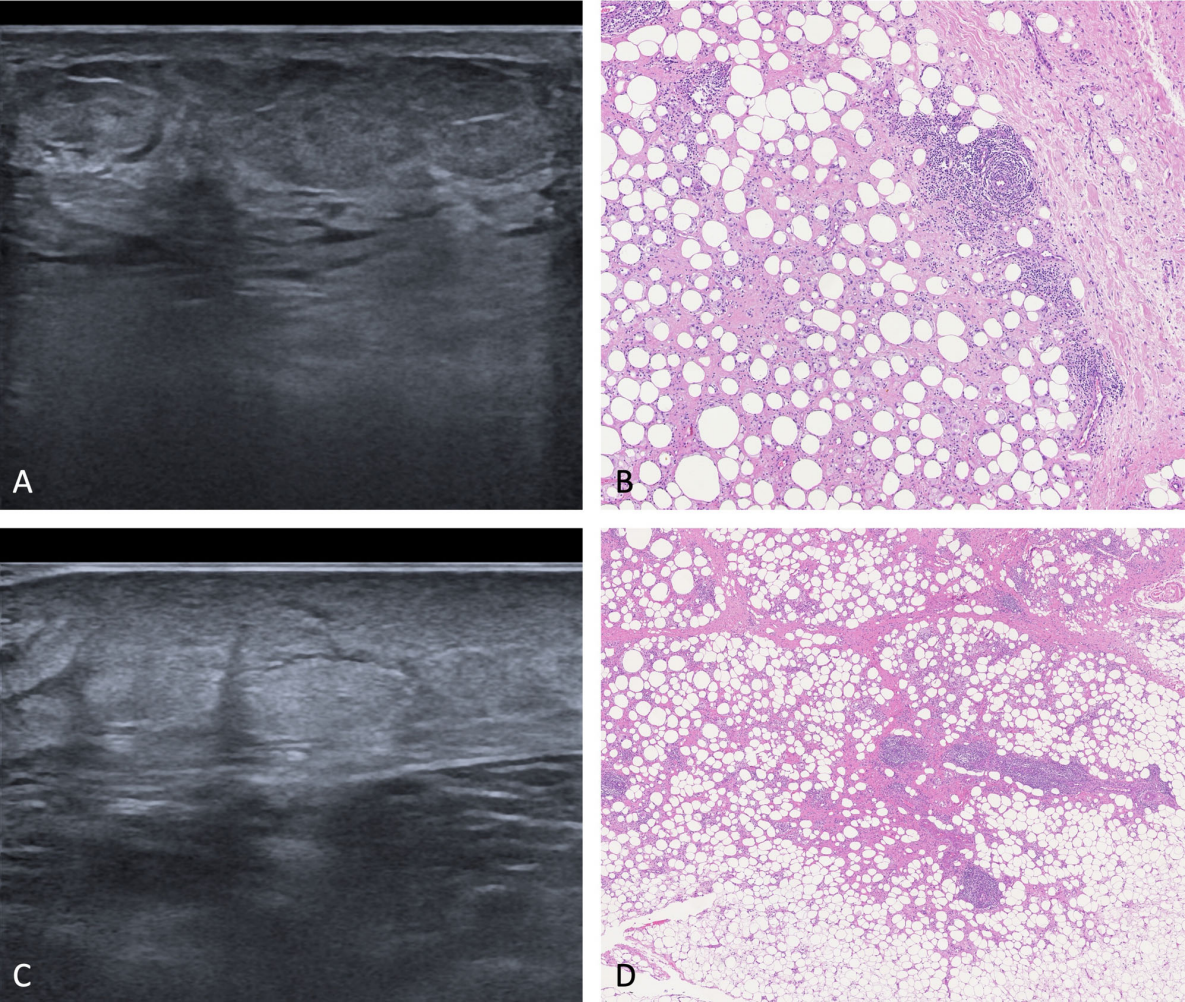
Mixed septal/lobular panniculitis. Ultrasonographic (A and C) features of mixed septal/lobular panniculitis showing blurrier septa with hyperechoic subcutaneous lobules focally blending together. On histology a greater infiltration of the subcutaneous lobules is appreciated (B and D) (H&E, 20x)

Subsequently, an incisional biopsy was taken, as histological examination is still considered the reference standard. Histological examination showed clear‐cut septal (4), and mixed septal/lobular panniculitis (1).

Considering histology as the gold standard, US allowed correct identification of five of five cases of EN. Moreover, reinforcing the concept of a sonographic‐histopathological correspondence, the histological finding of mixed septal/lobular panniculitis was noted in those cases showing blurriness of the subcutaneous septa on US.

Cutaneous ultrasound is a relatively novel, dermatological diagnostic imaging technique, offering noninvasive, rapid assessment in a wide variety of clinical settings.^2^

Recently, Romani et al. studied the role of high‐frequency dermatologic ultrasound in distinguishing mainly septal from mainly lobular panniculitis. A multicentric, prospective, inter‐ and intra‐rater agreement study was conducted, involving 64 individuals, of whom 32 had a final diagnosis of EN. Sensitivity and specificity of dermatologic US for the diagnosis of lobular panniculitis were assessed to be 85.19% and 88.57%, respectively.^3^

Proposed criteria for predominantly septal panniculitis were (1) septal thickening and hypoechogenicity (thickness ≥1 mm in three or more septa), preferably in proximity of not compressible fat lobules; (2) frequent hyperechogenicity of adipose lobules, and (3) an increase in Doppler flow. Overall, a “jigsaw” appearance was described.

Conversely, predominantly lobular panniculitis was defined by (1) the lack of features typical of septal panniculitis; (2) hyperechoic, blurry adipose lobules; (3) with no increase in Doppler flow.

Hyperechoic muscle with a hypoechoic, thickened fascia, hyperechoic calcifications, as well as anechoic pseudo cystic areas of fat necrosis were additional, albeit not exclusive, criteria for lobular panniculitis.^3^

Maldonado et al. reported a case of gouty panniculitis, highlighting isoechoic and hyperechoic masses in the subcutis with a posterior acoustic shadow artifact and an inconstant, surrounding hypoechoic rim. These features were associated with a lobular‐type panniculitis on histology.^4^

In our case series, the core findings of septal panniculitis were confirmed to be hypoechoic, not compressible septa, in the context of a hyperechoic, lobular hypodermis. Furthermore, decreased prominence/blurriness of septal appearance may be indicative of lobular involvement, albeit minor.

From a practical perspective, where a dedicated radiology/sonography service is not readily available, dermatological US may spare EN the necessity for histological confirmation, even in clinically dubious cases.

In conclusion, we reported five patients with EN and described the typical US features of septal panniculitis, that is, the presence of hypoechoic, not compressible septa, in the context of a hyperechoic hypodermis. Our findings are in line with those published by Romani et al.^3^ and further underscore the importance of sonographic‐pathological correlation.

To the best of our knowledge, this is one of very few reports documenting EN ultrasonographic features. Although no definitive recommendations can be formulated, ultrasonography may offer a practical, noninvasive approach to diagnostically challenging cases of EN, potentially sparing the patients the discomfort of an incisional biopsy.

## CONFLICT OF INTEREST

The authors declare no conflict of interest.
